# Physical properties of metal-doped zinc oxide films for surface acoustic wave application

**DOI:** 10.1186/1556-276X-7-25

**Published:** 2012-01-05

**Authors:** Sang-Hun Nam, Sang-Jin Cho, Jin-Hyo Boo

**Affiliations:** 1 Department of Chemistry and Institute of Basic Science, Sungkyunkwan University, Suwon, 440-746, South Korea

**Keywords:** MZO, surface acoustic waves, RF magnetron sputter, ZnO, piezoelectric devices

## Abstract

Metal-doped ZnO [MZO] thin films show changes of the following properties by a dopant. First, group III element (Al, In, Ga)-doped ZnO thin films have a high conductivity having an n-type semiconductor characteristic. Second, group I element (Li, Na, K)-doped ZnO thin films have high resistivity due to a dopant that accepts a carrier. The metal-doped ZnO (M = Li, Ag) films were prepared by radio frequency magnetron sputtering on glass substrates with the MZO targets. We investigated on the optical and electrical properties of the as-sputtered MZO films as dependences on the doping contents in the targets. All the MZO films had shown a preferred orientation in the [002] direction. As the quantity and the variety of metal dopants were changed, the crystallinity and the transmittance, as well as optical band gap were changed. The electrical resistivity was also changed with changing metal doping amounts and kinds of dopants. An epitaxial Li-doped ZnO film has a high resistivity and very smooth surface; it will have the most optimum conditions which can be used for the piezoelectric devices.

## Introduction

ZnO is a group II-VI compound semiconductor that has a hexagonal wurtzite structure. ZnO has typical n-type semiconductor properties because of a nonstoichiometric defected structure and electrical properties according to chemical composition changes. ZnO has piezoelectric properties, wide band gap (3.37 eV), etc. Metal-doped ZnO [MZO] thin films show changes of the following properties by a dopant [1]: First, group III element (Al, In, Ga)-doped ZnO thin films have a high conductivity having an n-type semiconductor characteristic [2,3]. Second, group I element (Li, Na, and K)-doped ZnO thin films have a high resistivity due to a dopant that accepts a carrier [4]. In the case of the group II-VI oxide semiconductor, oxygen defect, chemical composition, and impurities have significant influences on physical properties such as electrical resistivity, piezoelectricity, and structure. The change of property in ZnO had been reported. It was found that Li atoms in Li-doped ZnO films were involved in the substitution for Zn atoms; they acted as acceptors that compensate the excess Zn atoms [5]. The ZnO film is not only a strong *c*-axis-oriented crystalline structure, but has also a high resistivity of above 10 Ω cm when used for the piezoelectric device applications. We mainly studied on the effects of deposition conditions on the structural and electrical properties of ZnO films that will be applied as piezoelectric devices.

## Experimental detail

Two kinds sputtering targets were prepared by mixing with zinc oxide and lithium chloride powders, and zinc oxide and silver nitrate powders where the ratio of dopants was increased from 0 to 10 wt.% by an interval of 2 wt.%. MZO films were deposited on glass substrates at room temperature with a radio frequency [RF] power of 150 W at a target-to-substrate distance of 70 mm. The thickness of the MZO films was up to 500 nm on the glass substrates for optical measurements. The crystal structure, microstructure, and the thickness were observed using X-ray diffraction [XRD] and a scanning electron microscope. Atomic force microscopy was used to examine the surface roughness. X-ray photoelectron spectroscopy and energy dispersive X-ray spectroscopy were also utilized to analyze the chemical ratio of the MZO films. The electrical resistivity was measured by a four-point probe method.

## Results and discussion

**Figure 1:**
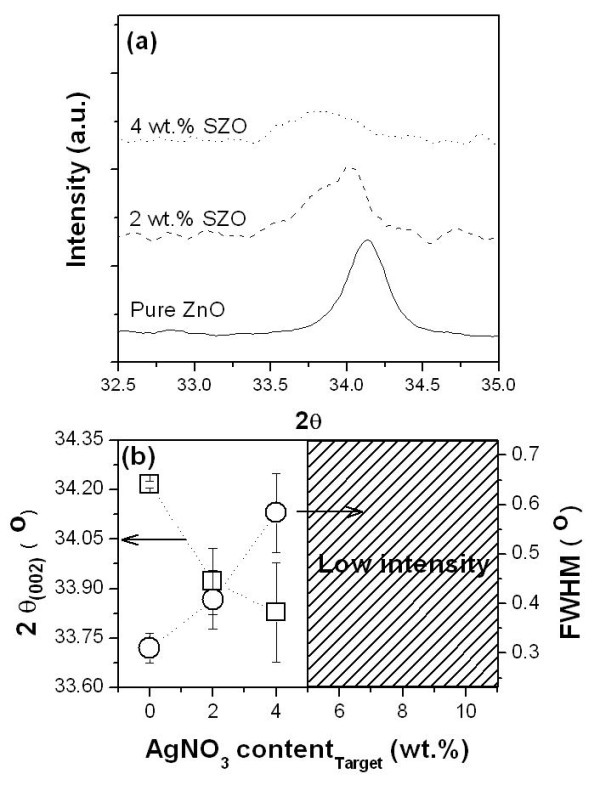
**Structural characteristics of SZO films**. (**a**)The high-resolution XRD graphs of SZO films and (**b**) the FWHMs and the intensity of the (002) diffraction peaks (square: 2*θ*; circle: FWHM).

**Figure 2:**
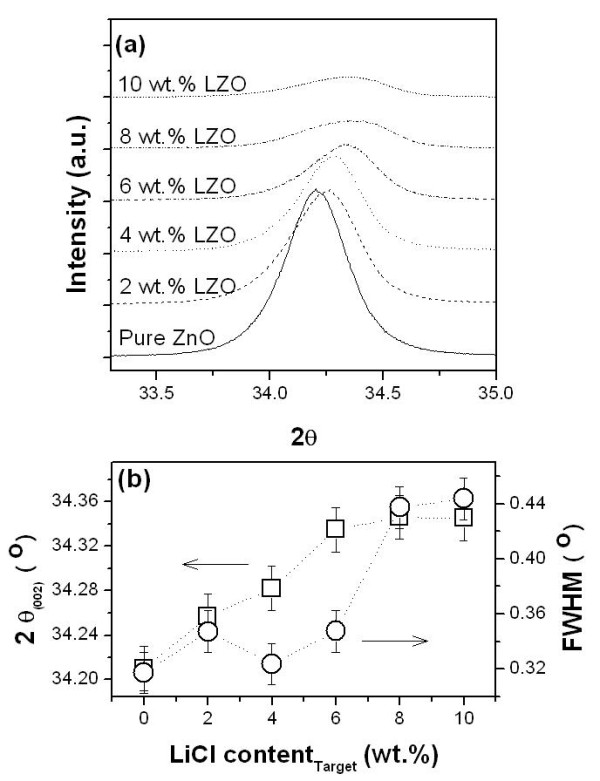
**Structural characteristics of LZO films**. (**a**) The high-resolution X-ray (002) diffraction peaks of both pure ZnO and LZO films and (**b**) the (002) peak positions and FWHM values of the LZO films (square: 2*θ*; circle: FWHM).

Typical XRD patterns of the MZO films were observed only in the (002) peaks at 2*θ *≈ 34°, indicating that all of the obtained MZO films had a preferred orientation with the *c*-axis. Figure 1a shows the high-resolution XRD graphs of silver-doped ZnO [SZO] films. The (002) diffraction peaks of the SZO films are observed at 2*θ *= 34.21° to 33.83°; the (002) diffraction peaks are shifted to lower 2*θ *values with increasing doping amounts. The reason for the result is that the lattice parameter of the SZO films was increased in the *c*-axis with the increasing Ag dopant such that the large Ag ions (122 pm) were substituted for the Zn ions (72 pm) in the SZO crystal. The full width half maximums [FWHMs] and the intensity of the peaks were carried out to evaluate the crystalline quality of the films as shown in Figure 1b. As a result, the FWHMs of the (002) peaks decreased with increasing Ag dopant (0.3093°, 0.4077°, and 0.5835°). This means that the crystallinity decreased considerably with increasing Ag dopant in the SZO films while a large silver ion substituted into a zinc ion site. The SZO films with poor crystallinity are hard to apply in piezoelectric devices. High-resolution X-ray (002) diffraction peaks of both pure ZnO and Li-doped ZnO [LZO] films are shown in Figure 2a. As the amount of the Li dopant increased, the intensity and angle of the (002) diffraction peak are also decreased and increased, respectively. These results indicate that the lattice constants of the ZnO crystals are gradually decreased due to Li substitution. In detail, with the increasing amount of Li dopant, an asymmetric (002) diffraction peak that is attributed to the LZO thin film formation appeared, and the diffraction angle is also shifted toward a high degree from 34.21° to 34.35°. This suggests that the lattice constants in the *c*-axis of the ZnO crystal (or films) decrease with the substitution of Li ion into Zn sites. Many researchers reported that most of the Zn sites were substituted by a doping ion of a larger size than the Zn ion. On the contrary, it was reported that dopants can be substituted or inserted because the doping ion was smaller than the Zn ion [6,7]. This is in good agreement with our data. As Li-doped amounts increase, the intensities of ZnO (002) peaks decrease, and their FWHM value increased up to 0.444°. The (002) peak positions and FWHM values of the LZO films were shown in Figure 2b.

**Figure 3:**
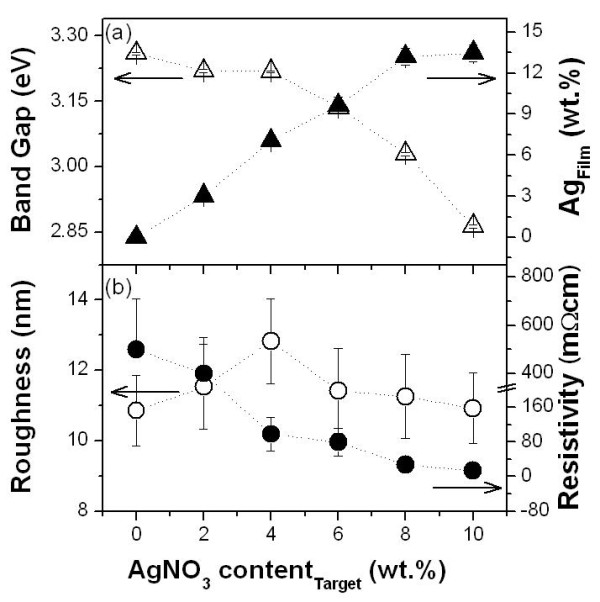
**Properties of the SZO films**. (**a**) The optical band gap and silver quantity and (**b**) the RMS roughness and electrical resistivity in the SZO films. Open triangles denote the dopant quantities in the MZO films, while solid triangles, the dopant amounts in the MZO ceramic targets. Open circles denote the RMS roughness, and solid circles, the electrical resistivity.

**Figure 4:**
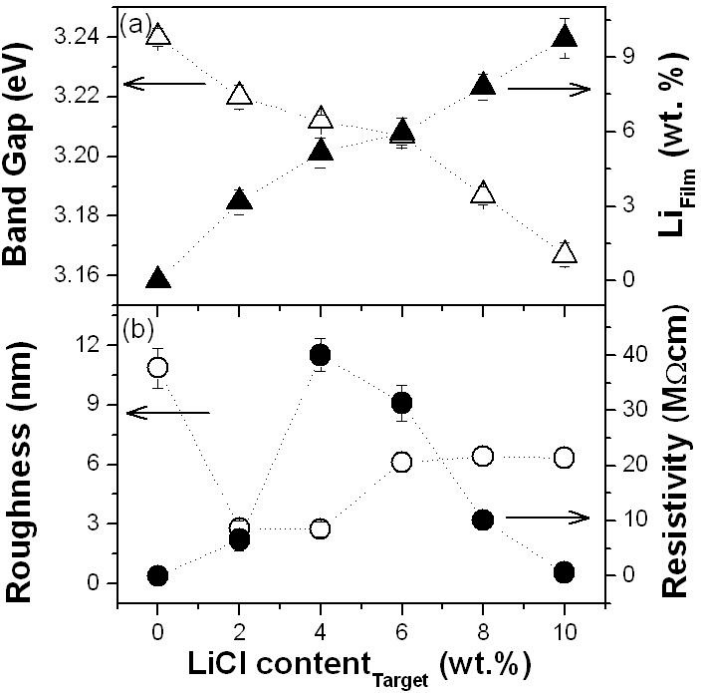
**Properties of the LZO films**. (**a**) The optical band gap and lithium quantity and (**b**) the RMS roughness and electrical resistivity in the LZO films. Open triangles denote the dopant quantities in the MZO films, while solid triangles, the dopant amounts in the MZO ceramic targets. Open circles denote the RMS roughness, and solid circles, the electrical resistivity.

Figures 3a and 4a show the dopant quantity and optical band gap of the MZO films. The dopant compositions were calculated using a high-resolution XP spectra, and the optical band gap could be obtained by plotting *α*^2 ^vs. *hν *(*α *is the absorption coefficient and *hν *is the photon energy) and extrapolating the straight-line portion of this plot to the photon energy axis [7]. The left *y*-axis of Figures 3a and 4a show the variation of the optical band gap as a function of dopant contents. It showed that the optical band gap narrows with increasing dopant contents because aggregated doping metals substitute into the zinc sites. The dopant quantities in the MZO films are linearly increased with the dopant amounts in the MZO ceramic targets (solid triangle in Figures 3 and 4a).

MZO films are required to have a high electrical resistivity and smooth surface morphology for piezoelectric device application. To find film uniformity, we measured the surface root mean square [RMS] roughness. Figures 3b and 4b show the RMS roughness (open circle) and electrical resistivity (solid circle) of the SZO and LZO films, respectively. As the surface roughness increases, electrical resistivity decreases with the addition of silver in the SZO films as shown in Figure 3b. The reason for this result is to aggregate doped silver with increasing Ag amounts. Low resistive SZO films are not appropriate for piezoelectric devices. Figure 4b shows that the surface RMS roughness decreased from 10.86 nm to 2. 743 nm with increasing Li contents from 0 to 4 wt.%. The surface RMS roughness is also retained regularly (about 6.4 nm) in the Li content range of 4 to 10 wt.%. The smoothest film is obtained from a LZO film with 4 wt.% Li content. In Figure 4b, between 0 and 4 wt.% Li contents, the electrical resistivity of the LZO films is increased up to 40 MΩ cm. Generally, ZnO thin films are n-type semiconductive metal-oxide thin films since the excess Zn or defective O atoms perform the part of a donor. These are induced so that the carriers contribute to the conductivity. However, resistivity only in a small amount of doped LZO thin films increases because Li is operated to reduce the carrier through an acceptor [8]. Therefore, we found that the resistivity increased until the formation of a 4 wt.% Li-doped ZnO thin film (107 Ω cm). As Li doping amounts are increased unduly (above 6 wt.%), resistivity increases because the excess Li atoms act as carriers.

## Conclusions

MZO films with various metal contents (Ag and Li of 0 to approximately 10 wt.%) were prepared by RF magnetron sputtering with especially designed ZnO targets. The structural, optical, and electrical properties of MZO films depended on the dopant content ratio in the target. The deposited MZO films have a preferred crystalline orientation in the [001] direction. However, the crystallinity and electrical resistivity of SZO films are not good for piezoelectric device applications. Highly oriented LZO thin films with 2 to 10 wt.% Li contents can be grown, and among them, the most crystalline LZO film with very smooth surface and high resistivity is observed from the 4 wt.% Li-doped LZO target; it will have the most optimum conditions for deposition which can be used for the piezoelectric devices.

## Competing interests

The authors declare that they have no competing interests.

## Authors' contributions

SHN and JHB conceived the study. SHN carried out the experiments and drafted the manuscript. SJC contributed to the analysis of the study. All authors were involved in revising the manuscript and approved the final version.
